# Alzheimer Disease Detection Studies: Perspective on Multi-Modal Data

**DOI:** 10.1055/s-0044-1800756

**Published:** 2025-04-08

**Authors:** Farzaneh Dehghani, Reihaneh Derafshi, Joanna Lin, Sayeh Bayat, Mariana Bento

**Affiliations:** 1 Biomedical Engineering Department, University of Calgary, Canada; 2 Computer Science Department, University of Calgary, Canada; 3 Geomatics Engineering Department, University of Calgary, Canada; 4 Electrical and Software Engineering Department, University of Calgary, Canada; 5 Hotchkiss Brain Institute, Cumming School of Medicine, University of Calgary, Canada

**Keywords:** Alzheimer's Disease, Signals and Sensors, Medical Imaging, Electronic Medical Records, Computer-aided Diagnosis

## Abstract

**Objectives** : Alzheimer's Disease (AD) is one of the most common neurodegenerative diseases, resulting in progressive cognitive decline, and so accurate and timely AD diagnosis is of critical importance. To this end, various medical technologies and computer-aided diagnosis (CAD), ranging from biosensors and raw signals to medical imaging, have been used to provide information about the state of AD. In this survey, we aim to provide a review on CAD systems for automated AD detection, focusing on different data types: namely, signals and sensors, medical imaging, and electronic medical records (EMR).

**Methods** : We explored the literature on automated AD detection from 2022-2023. Specifically, we focused on various data resources and reviewed several preprocessing and learning methodologies applied to each data type, as well as evaluation metrics for model performance evaluation. Further, we focused on challenges, future perspectives, and recommendations regarding automated AD diagnosis.

**Results** : Compared to other modalities, medical imaging was the most common data type. The prominent modality was Magnetic Resonance Imaging (MRI). In contrast, studies based on EMR data type were marginal because EMR is mostly used for AD prediction rather than detection. Several challenges were identified: data scarcity and bias, imbalanced datasets, missing information, anonymization, lack of standardization, and explainability.

**Conclusion** : Despite recent developments in automated AD detection, improving the trustworthiness and performance of these models, and combining different data types will improve usability and reliability of CAD tools for early AD detection in the clinical practice.

## 1. Introduction

Alzheimer's Disease (AD) is an irreversible and progressive neurodegenerative brain disorder that causes gradual memory loss, cognitive impairment, and emotional distress [ 1 ]. Currently, there is no cure for AD; therefore, early diagnosis provides the best prognosis and slows the progression [ 2 ]. Various medical technologies have been used for early and accurate AD detection. Biosensors are analytical devices that measure biological reactions and transfer them into quantifiable signals. Electrochemical biosensors are advantageous in monitoring Amyloid-β (Aβ) as the main biomarkers for AD detection. Behavioral detection sensors, subtypes of biosensors, are used to monitor the activity of older adults [ 3 ]. Wearable sensors have unveiled novel avenues for real-time monitoring of patients and ubiquitous access to vital patient data. Inertial measurement unit (IMU) sensors, including accelerometer, gyroscope, and magnetometer, play a pivotal role in AD detection. By calculating the angle, velocity, gravitational force, and orientation of patients with AD, IMU can be used for gait analysis and daily-life activity monitoring of these patients [ 4 ]. Raw signals such as electroencephalogram (EEG) signals or radio frequency (RF) signals can be utilized for AD detection in a noninvasive, comfortable way. Alteration in the regional cerebral blood flow (rCBF) is one of the leading causes of abnormalities in EEG signal of patients with AD [ 5 ]. Neuroimaging modalities, such as MRI and Positron Emission Tomography (PET), play a significant role in AD detection. MRI imaging allows studying neurodegeneration and pathological changes of the brain, while PET modality monitors the brain's glucose intake or amyloid protein. Aside from these data types are EMR, consisting of demographics, biomarkers, and biosignature [ 6 ].

## 2. Methods

**Figure 1.**
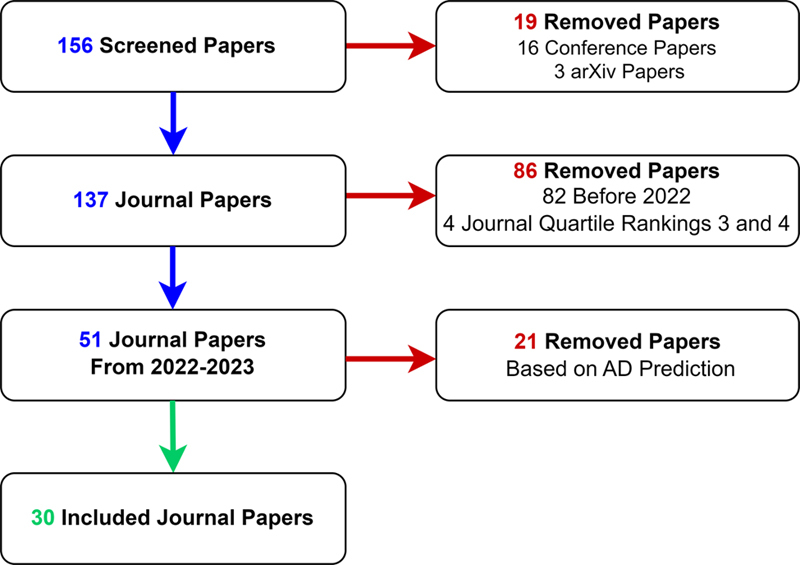
The screening process of the literature review.

This survey needs to introduce and assess different data types used for CAD-based AD detection based on Machine Learning (ML) and Deep Learning (DL) methods. For our literature review, we used Scopus, PubMed, IEEE Xplore, and ScienceDirect as online databases to search for papers. For the search query, we identified words or combination of words to look for in the title and abstract of articles, which are as follows: “Alzheimer's Disease” AND “Detection, Prediction” AND “Machine Learning, Deep Learning, Artificial Intelligence” AND “Imaging, health records, sensor data, wearable sensors, Gait Analysis, Sleep monitoring, Physiological Sensors, IoT (Internet of Things) in Healthcare” AND “MRI, PET, Accelerometer, Gyroscope, EEG, RFID (Radio-Frequency Identification), Biosensor”. After importing 156 articles, we narrowed the selection to 30 based on the following criteria. Only journal articles published between 2022 and 2023 with journal quartile rankings 1 and 2 were included, as this timeframe ensures the incorporation of recent research findings while prioritizing publications from journals with academic impact. We included papers with established results and discussions, mitigating the inclusion of papers with preliminary results/findings. The approach is illustrated in Figure 1 . In this figure, a comprehensive explanation of the inclusion and exclusion criteria is included, aiming to further understand dementia studies using multiple data modalities.

We performed a comprehensive literature review in the included papers considering various stages, ranging from preprocessing, feature extraction, and classification to model evaluation. The aim is to provide an overview of the methods used at each stage of the machine learning pipeline. Data plays a pivotal role in the performance of AI-based systems, and applying appropriate preprocessing methods may impact outcomes' reliability and performance. This step is crucial for electrophysiological signals such as EEG and MEG, which require noise filtering and artifact removal to produce clean data. Techniques such as independent component analysis (ICA) are often employed to identify and discard noise and artifacts, followed by signal normalization to reduce inter-subject variability.

In medical imaging studies, various preprocessing techniques are applied to improve the performance of models for AD detection. Intensity correction refers to mapping the intensities of pixels or voxels of an image onto a reference scale to ensure that similar structures in an image have similar intensities. Anterior commissure-posterior commissure (AC-PC) correction refers to aligning image geometry according to the AC and PC of the brain to ensure that the AC and PC of all image scans are in the same axial plane. Skull stripping refers to the process of removing the bone of the skull from image scans. Co-registration facilitates the comparison process between the voxel intensities of brain images of different subjects because of the same anatomical position of voxels across all image scans. Slice timing correction can be applied to correct differences in acquisition time between layers of a volume. Motion correction is applied to remove motion artefacts in brain images. Tissue segmentation is applied to measure the volume of tissue in each region, enabling the comparison between GM and WM, producing GM probability maps. These maps provide useful information about the spatial distribution of GM, which is impacted by neurodegeneration in the initial stages. The ML/DL models' performance depends on data fed into it; thus, increasing the amount of training data using augmentation techniques is of utmost importance. Data augmentation is also efficient when a dataset suffers from a class imbalance problem [ 7 ].

In medical imaging studies, input data management can be assorted into four categories according to the type of extracted features: voxel-based, slice-based, ROI-based, and patch-based. In the voxel-based approach the values of voxel intensities are used from all neuroimaging modalities. Slice-based techniques can be used to extract 2D slices from 3D brain scans to reduce the number of hyperparameters. In the ROI-based technique, instead of considering the whole brain, particular brain regions responsible for AD are extracted. In a patch-based approach, a 3D cube is extracted from brain scans to capture brain patterns related to AD [ 7 ].

In EMR studies, preprocessing and quality control stages should be applied. For example, irrelevant or redundant temporal data, such as duplicated date fields and variables related to protocols, should be removed from the dataset. Some fields containing negative values or string modifiers, such as less than or greater than, should be corrected [ 6 ].

**Figure 2.**
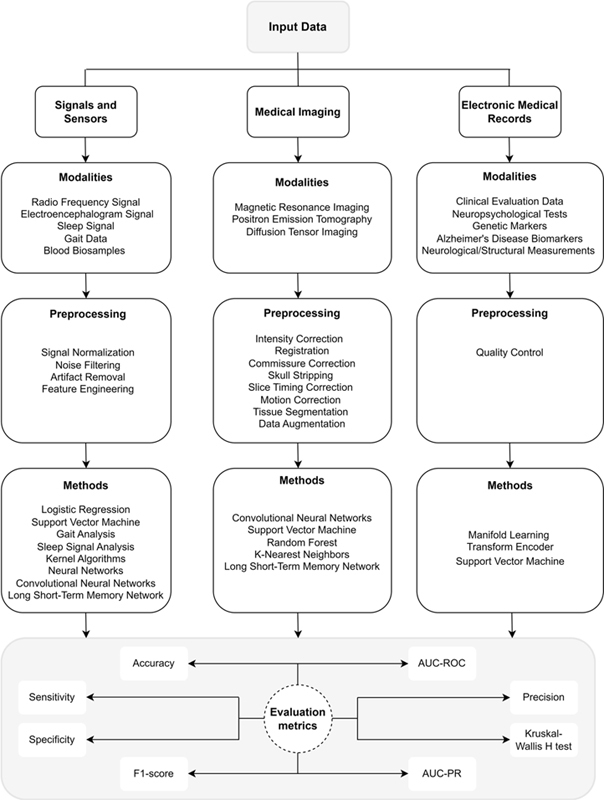
An overall perspective on various modalities and preprocessing stages applied for each data type, methods used for AD detection, as well as evaluation metrics applied for evaluating the performance of models developed for AD detection in our literature review.

Evaluation metrics serve as a comprehensive assessment framework, enabling researchers to quantify and compare the performance of various models and to ensure that the most effective techniques are identified for clinical application. The main evaluation metrics used by studies in our literature review are accuracy, sensitivity, specificity, precision, F1-score, AUC-ROC, AUC-PR, and Kruskal-Wallis H test [ 8 ]. The overall perspective on various modalities and preprocessing stages for different data types, together with the evaluation metrics discussed here, are illustrated in Figure 2 .

## 3. Results

This section presents our findings about available datasets, recent developments, preprocessing and learning methodologies, and evaluation metrics for automated AD detection based on the selected literature work.

### 3.1. Datasets

One key aspect of developing automated AD detection is the availability of public datasets. The characteristics and information about publicly available datasets used by the papers in this literature review are summarized in Table 1 .

- ADNI – Alzheimer's Disease Neuroimaging Initiative (ADNI 1 ) is a Longitudinal and multicenter study, comprising clinical, imaging, genetic, and biochemical biomarkers for AD diagnosis. ADNI is the most common dataset in our literature review.
- AIBL – The Australian Imaging, Biomarker and Lifestyle (AIBL 2 ) Study assesses the biomarkers, genetic factors, cognitive characteristics, and health and lifestyle factors contributing to AD progression. The main goal was to researching lifestyle factors and genetics for AD prediction.
- Alzheimer-MRI-dataset (Kaggle 3 ) – The dataset collects MRI data from various websites, enabling researchers to develop highly accurate models for classifying AD stages.
- OASIS – The Open Access Series of Imaging Studies (OASIS 4 ) comprises free neuroimaging datasets for future basic and clinical neuroscience developments. The OASIS dataset is comprised of four different datasets collected using different protocols with different aims.
- MIRIAD – The Minimal Interval Resonance Imaging in AD (MIRIAD 5 ) is a publicly available dataset that aims to consider the feasibility of MRI for clinical trials and AD treatment. The scans of many participants are collected at intervals of 2 weeks to 2 years.
- Dementia talk Bank – The Dementia Talk Bank 6 dataset is an expansive, multilingual collection, offering a rich array of transcripts, media files, protocols, and speech tasks in English, Spanish, Mandarin, and Taiwanese. It aggregates data from a myriad of international sources, each with a unique focus on specific patient groups and languages. With a subject pool exceeding 2,561 individuals, including various dementia stages and healthy controls, the dataset is instrumental for research into the linguistic indicators of dementia and the advancement of related diagnostic and therapeutic methodologies.

**Table 1.** Overview of various datasets and their characteristics used by studies in our literature review.

Dataset	Data type	Number of subjects	Patient diagnosis groups	Preprocessing
ADNI	Clinical Genetic MRI DTI fMRI PET Biospecimens	5,190	AD Normal Control MCI Early MCI Late MCI	Intensity Normalization Gradient Inhomogeneity Correction Co-registration
AIBL	Non-Imaging (Demographics, Medical History, Neuropsychology Scores, Blood Analysis) MRI PET	854	AD Normal Control	-
Kaggle	MRI	6,400	Mild Dementia Moderate Dementia Non-Dementia Very Mild Dementia	-
OASIS	MRI PET CT	3,059	AD Normal Control MCI	Skull Stripping Bias Field Correction
MIRIAD	MRI Demographics Psychological Data	69	AD Normal Control	-
Dementia Talk Bank	Transcripts and Media, Protocols and Speech Tasks	2,561	MCI AD Normal Control Parkinson's disease with MCI	-

### 3.2. Recent Developments in Automated AD Detection

#### 3.2.1. Signals and Sensors

In comparison to the entire signals and sensors applied for automated AD detection, EEG signal was the most common type (four out of eight studies worked on EEG signals). In [ 9 ], S-parameter data were obtained from 6 Radio Frequency (RF) antennas that were placed around the head of nine participants to noninvasively capture changes in the brain in the presence of AD pathology. Researchers in [ 10 ] introduced a novel directed graph for local texture feature extraction with EEG signals. In this paper, 16-channel EEG signal recordings of 12 AD patients and 11 healthy controls (HC) were collected. Researchers in [ 11 ] developed a fully automated technique for AD detection and discrimination of mild AD and mild cognitive impairment (MCI), based on the resting state EEG signal. In this research, two minutes recording of eye-open resting state activity of 21 subjects in three different stages was collected using 16 EEG channels. In [ 12 ], the aim was to report the preliminary evaluation of a self-driven AD multi-class discrimination approach based on a commercial EEG acquisition system using 16 channels.

In [ 13 ], seven wearable devices with a built-in IMU were used to collect gait data from 145 subjects for early diagnosis of AD, including seven gait experiment paradigms and multilevel subtasks. In [ 14 ], an overnight recording of movements associated with sleep combined with advanced signal processing and AI was proposed for MCI detection. In this paper, the SleepMoveTM continuously recorded data of 40 participants. In [ 15 ], researchers developed a diagnostic model for AD based on federated learning (FL) and hardware acceleration using blood biosamples provided by ADNI. In [ 16 ], researchers applied highly informative BiLSTM and CNN features of EEG signal for AD diagnosis, comprising 24 healthy elderly people and 24 subjects diagnosed with AD, each EEG signal captured both temporal brain activity patterns and regional brain information for AD diagnosis.

#### 3.2.2. Medical Imaging

Most studies investigated in this review are based on medical imaging applied MRI scans for automated AD detection (17 out of 20 studies). In contrast, only three out of 20 studies used PET scans. All the studies discussed here are based on CNN and have various architectures. In [ 2 ], the aim was to develop an explainable, end-to-end DL-based framework to exploit global, local, and spatial information from MRI images and identify and visualize key areas contributing to AD diagnosis. They used ADNI dataset to classify AD from normal control (NC), and progressive MCI from stable MCI. Researchers in [ 17 ] developed a DL-based framework to extract and fuse multiple spatial-scale features for AD classification and provide an attention block to visualize and interpret AD biomarkers using ADNI. In [ 18 ], researchers developed a DL-based algorithm using three different classifiers for AD diagnosis using ADNI and MIRIAD. In [ 19 ], researchers proposed a 2D CNN for early AD and MCI detection from MRI provided by ADNI. Their system applies to other diseases, time-efficient, and effective for limited datasets. In [ 20 ], researchers aimed to develop a 3D CNN-based method for AD and MCI classification by combining supervised learning and unsupervised contrastive learning algorithms using ADNI.

In [ 21 ] the aim was to develop an end-to-end DL approach based on Re-CNN and extract deep semantic features and morphological metrics to classify AD from MRI images, using ADNI for training and validation, and AIBL dataset for testing. Researchers in [ 22 ] developed a DL-based approach based on multi-scale CNN and channel attention mechanism to extract and fuse features of segmented gray matter (GM) and white matter (WM) using ADNI. Researchers in [ 23 ] proposed a DL-based approach for classification of different stages of AD when using small datasets and applied ADASYN oversampling method to overcome the problem of unbalanced number of classes using MRI from Kaggle. In [ 24 ], researchers developed a 2D DL-based classification approach for AD diagnosis and improved the transparency of their method by applying Grad-CAM++. They used ADNI to develop the model and AIBL to ensure model generalizability. In [ 25 ], the aim was to compare 2D and 3D DL-based approaches for AD and MCI classification based on PET images from ADNI. In [ 26 ], researchers developed a DL-based approach for classification of different AD stages using multi-modality MRI (MRI and DTI) from ADNI dataset. The aim of [ 27 ] was to develop a 2D CNN framework for AD detection using 18FDG-PET images provided by ADNI.

In [ 28 ], the aim was to propose a cascaded CNN-LSTM model to classify 3D MRI from ADNI for AD diagnosis to reduce the computational cost of 3D CNN and employ knowledge distillation to enhance accuracy in small samples. Researchers in [ 1 ] developed a DL framework based on MobileNet with ImageNet weights to classify different stages of AD using brain MRI scan. They used ADNI dataset to classify AD, MCI, early MCI (EMCI), late MCI (LMCI), and NC. Researchers in [ 29 ] developed three different DL-based models based on different combinations of features of GoogLeNet and Dense-121 models as well as handcrafted features to detect different stages of AD progression from MRI provided by Kaggle and ADNI. Researchers in [ 30 ] developed a 2D DL-based framework combining CNN and Transformer to extract features and perform feature fusion to classify AD and MCI using MRI images. They used ADNI for training and OASIS for generalizability of their model. The aim of [ 31 ] was to develop a DL-based framework that extracts whole brain Jacobian domain features at the subject level to train 3D CNN for AD diagnosis using ADNI. In [ 32 ], researchers proposed a method to compare the performance of 3 types of patch-wise sampling methods based on cubic image patches, cuboid image patches, and ROI-based image patches, for 3D CNN-based AD classification using ADNI. In [ 33 ], researchers proposed a method to compare vision transformers (ViT) and a CNN-based model for AD classification on FBB PET images. In [ 34 ], the aim was to develop a ML framework that combines a CNN and the K-nearest neighbor (KNN) to extract and classify informative features from MRI images provided by Kaggle, detecting four different stages of AD.

#### 3.2.3. Electronic Medical Records

Among all studies considered here, very few of them (only two) were based on EMR for AD detection. In [ 35 ], authors fused brain MRI scans with genetic data to develop a DL framework for AD classification using ADNI dataset. They applied a model, called IGNet, composed of a 3D CNN network as an imaging channel and a Transform encoder as a genetic channel. In [ 6 ], researchers developed an unsupervised learning algorithm to classify different stages of AD using EMR. They predicted AD progression by applying various manifold learning methods along with an autoencoder-based embedding of cognitive tests, CSF, and other biomarkers.

### 3.3. Preprocessing and Learning Methodologies for Automated AD Detection

#### 3.3.1. Signals and Sensors

Wearable devices, which track movement, vitals, and sleep patterns, produce continuous, large data streams that can often be noisy due to motion artifacts or environmental variabilities. The preprocessing of these datasets starts with the application of filters to remove noise. The filters applied to wearable device data typically include low-pass filters to remove high-frequency noise and high-pass filters to eliminate signal drift, along with band-pass filters that preserve frequencies relevant to human activity and physiological measurements. The data is then segmented to identify meaningful patterns corresponding to different activities or states. Normalization of wearable data is crucial to ensure that variations in sensor sensitivity or user behavior do not affect the analysis. This process is augmented by feature engineering, where significant features are extracted from the time and frequency domains to simplify the data for analysis. Wearables may sometimes fail to collect data, resulting in gaps that are filled using imputation techniques to maintain the continuity of the dataset. Data from multiple sensors is integrated through data fusion techniques to create a comprehensive dataset that accurately represents the patient's condition. Calibrating the devices ensures that sensor readings remain accurate and consistent over time. Given the extensive volume of data from wearable devices, techniques like Principal Component Analysis (PCA) are utilized to reduce the data volume, facilitating more efficient computation without substantial information loss.

In our review, we delve into a range of innovative methodologies that harness various sensor technologies to enhance AD detection. In [ 9 ], S-parameter data captured by radio-frequency (RF) antennas placed around the head are analyzed using Logistic Regression and Support Vector Machines (SVM) to discern brain changes indicative of AD pathology. In the realm of EEG, multiple studies [ 10 11 12 , 16 ] have pioneered techniques to distill complex neurological signals into diagnosable patterns. Authors in [ 10 ] developed a directed graph methodology for local texture feature extraction, combined with Time-Frequency Representation (TFR) and iterative neighborhood component analysis. Another [ 11 ] utilizes EEG for AD detection by applying ML models to resting-state signal analysis. The fusion of high-informative features from BiLSTM and CNN models [ 16 ] illustrates the potential of DL in interpreting EEG signals for AD diagnosis, a technique that benefits from the rich temporal and spatial information contained within the EEG data.

The application of wearable sensors in gait assessment [ 13 ] offers a unique vantage point, capturing the intricacies of movement correlated with AD severity levels. Using devices equipped with accelerometers, gyroscopes, and magnetometers, multilevel gait analysis is conducted through ML ensemble methods. Sleep signal analysis [ 14 ] using a device that records movements associated with sleep emerges as a pivotal method for MCI detection. Neural Networks and Kernel algorithms are employed to analyze the data, providing a method that could be used in a home environment for early AD detection. The FL model [ 15 ] stands out by offering a new approach for privacy-preserving AD detection. By utilizing blood biosample datasets, this approach leverages hardware acceleration to enhance the diagnostic models, demonstrating that non-imaging biomarkers can be as critical as imaging in AD detection.

#### 3.3.2. Medical Imaging

Various Convolutional Neural Network (CNN) architectures are applied for AD detection in our literature review, including ResNet [ 2 , 18 , 22 , 24 , 28 ], MobileNet [ 1 ], VGGNet [ 30 , 32 , 33 ], LeNet [ 19 ], GoogLeNet [ 29 ], DenseNet [ 24 , 28 , 29 ], U-Net [ 20 ], Reparametrized-CNN (Re-CNN) [ 21 ], Jacobean domain-CNN (JD-CNN) [ 31 ], EfficientNet [ 24 ], and XceptionNet [ 25 ]. In some studies, CNN features are combined with hand crafted features, such as Discrete Wavelet Transform (DWT), Local Binary Pattern (LBP), and Garay Level Co-occurrence Matrix (GLCM) [ 29 ]. In one paper [ 29 ], PCA was applied to reduce dimensionality of features extracted by CNN model, contributing to the enhancement of model performance. In another research [ 20 ], histogram equalization, sharpening, and flipping are reported as the most effective data transformation methods to enhance model performance.

In some studies, the attention mechanism of CNN is introduced to the proposed model to improve its performance [ 2 , 17 , 22 , 30 , 33 ]. Self-attention mechanism, by giving weight to each part of an image, provides information about the importance of each part in the decision-making process. Adding self-attention mechanism to the CNN model provides feasibility of capturing regional, global, and spatial features from images [ 2 ]. For automatic classification of CNN models, different classifiers, such as SVM, Random Forest (RF), and Softmax are applied, and Softmax results in higher performance as compared to others [ 18 ]. In other studies, KNN [ 34 ] and LSTM [ 28 ] are applied for the classification task. Due to the black box nature of DL models, some studies added explanation methods, such as Grad-CAM, to improve explainability and transparency of their models [ 2 , 17 , 20 , 23 , 24 , 30 ].

#### 3.3.3. Electronic Medical Records

**Figure 3.**
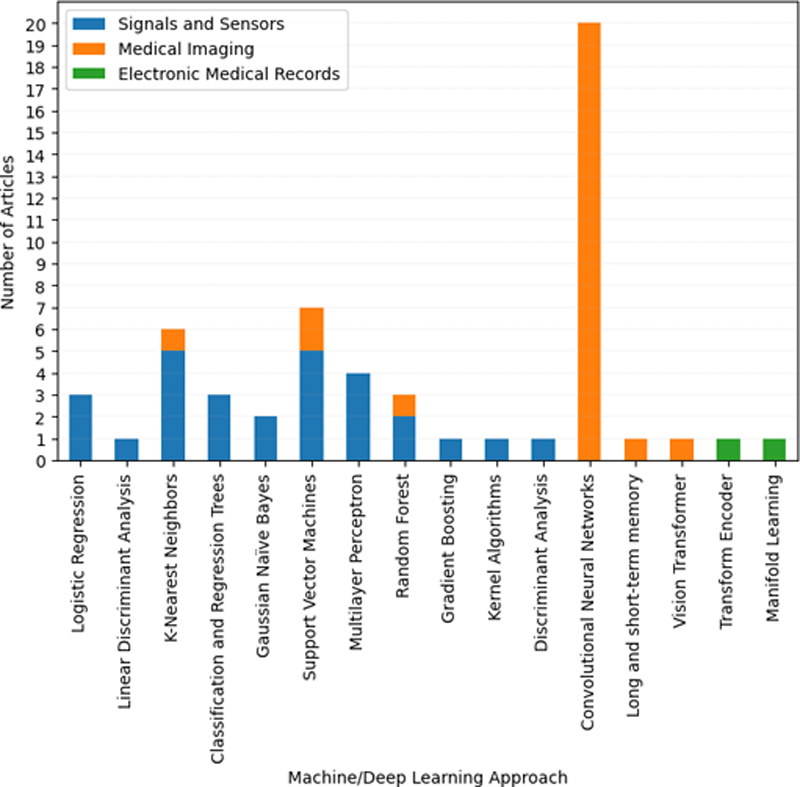
The distribution of different ML/DL techniques used by studies in our literature review across three data modalities.

Various manifold learning methods, including spectral embedding, multidimensional scaling (MDS), Isomap, t-Distributed Stochastic Neighbor Embedding (t-SNE), Uniform Manifold Approximation and Projection (UMAP), and a sparse denoising autoencoder are applied on biomarkers to assess their value in analyzing AD detection [ 6 ]. Of the methods applied, t-SNE and autoencoder embeddings resulted in higher performance for AD classification using SVM. In another paper [ 35 ], medical imaging and genetic sequencing are combined to detect AD automatically, where attention mechanism is applied for the genetic channel. Summary of the ML and DL methods used by studies in our literature review is presented in Table 2 . The distribution of different ML and DL techniques used by studies in our literature review across three data modalities, namely, signals and sensors, medical imaging, and EMR, is illustrated in Figure 3 .

**Table 2.** An analysis of different Machine/Deep Learning methods for AD detection used by studies in our literature review.

Data Modality	Data Type	ML approach	REFERENCE
Signals/Sensors	Radio Frequency	Logistic Regression (LR) Linear Discriminant Analysis (LDA) K-Nearest Neighbors (KNN) Regression Trees Gaussian Naïve Bayes (GNB) Support Vector Machines (SVM)	[ 9 ] [ 9 ] [ 9 ] [ 9 ] [ 9 ] [ 9 ] [ 9 ]
	Electroencephalogram (EEG)	KNN Multilayer Perceptron (MLP) RF SVM KNN Discriminant Analysis (DA)	[ 10 ] [ 12 ] [ 16 ] [ 16 ] [ 16 ] [ 16 ]
	Resting State EEG	LR SVM	[ 11 ] [ 11 ]
	Gait Data	Decision Tree (DT) GNB KNN SVM Random Forest (RF) Gradient boosting MLP	[ 13 ] [ 13 ] [ 13 ] [ 13 ] [ 13 ] [ 13 ] [ 13 ]
	Sleep Signal	MLP Kernel algorithms	[ 14 ] [ 14 ]
	Blood Bio-Samples	KNN SVM DT LR MLP	[ 15 ] [ 15 ] [ 15 ] [ 15 ] [ 15 ]
Medical Imaging	MRI	Residual Self-Attention	[ 2 ]
	MRI	CNN	[ 1 , 2 , 17 18 19 20 21 22 23 24 , 28 29 30 31 32 , 34 ]
	PET	CNN	[ 25 , 27 ]
	DTI and sMRI	CNN (with SVM classifier)	[ 26 ]
	PET	Vision Transformer	[ 33 ]
Electronic Records	Genetic Sequencing	Transform Encoder	[ 35 ]
	Clinical Evaluation	Manifold Learning	[ 6 ]

## 4. Discussion

In this paper, we surveyed recent developments in AD detection using ML and DL techniques by focusing on signals and sensors, medical imaging, and EMR data types. Most studies we investigated in this survey are based on medical imaging (20 out of 30), and especially on MRI (17 studies used MRI), as compared to other modalities. Due to MRI's strong tissue contrast and spatial resolution, structural changes associated with brain cells are immediately identifiable in this modality. Further, MRI imaging is more affordable and economically reasonable than other imaging modalities, especially PET [ 31 ]. However, the accessibility of MRI is limited by the requirement for specialized equipment and facilities, which may not be available in all clinical settings. This can pose challenges in the widespread adoption of MRI for AD detection, particularly in resource-limited environments. Conversely, the potential of wearable sensors, offering continuous real-time data, remains underexplored in AD detection. Of the entire papers considered here, eight papers were based on signals and sensors. Yet, the reliability of data from wearable sensors depends on the device's accuracy and the consistency of data collection protocols, which need to be standardized to improve comparability across studies. As for EMR, most papers are based on AD prediction rather than detection, thus, the number of papers in this context is marginal. Only two out of 30 papers in our literature review were based on EMR data type. EMR offers a rich source of longitudinal patient data, making it highly relevant for tracking disease progression and treatment outcomes. However, the feasibility of using EMR for AD detection is contingent upon the availability of comprehensive and standardized records, as well as robust methods for data anonymization to protect patient privacy.

According to Figure 3 , while most of the studies in our literature review based on signals and sensors applied ML techniques for automated AD detection, there are few studies that used DL for this aim. This could be due to some reasons, one of which is that the quality of data plays an important role when applying DL models. However, obtaining high quality signal data is difficult due to so much noise and variability. Further, having a high-performance DL model depends on the amount of data used, which is not always attainable in signals and sensors to obtain large datasets. In contrast to signals and sensors, all the studies based on medical imaging in our literature review used CNN for this purpose. This great tendency of researchers working on medical imaging to apply CNN for automated AD detection is due to the high performance, end-to-end learning, and minimized computation of this algorithm. As for EMR data type, both ML and DL techniques are applied for AD diagnosis.

Despite the vast number of review papers in the field of automated AD detection, very few of them focused on various data types. Specifically, signals and sensors and EMR modalities are not broadly appeared in review papers for AD detection. The most recent research [ 36 ] investigated state-of-the-art Artificial Intelligence methods for AD detection based on neuroimaging and signals and sensors data types. However, in our survey, papers based on different data types, ranging from medical imaging and wearable devices to EMR, are considered for AD diagnosis. Although researchers in [ 37 ] provided a survey to review the most recent advancements in automated AD detection, they just focused on papers that used DL methods for this aim. In contrast, our review is not restricted to the application of DL methods in AD diagnosis. We considered various papers wherein different ML and DL methods were applied.

In the following section, we intend to discuss challenges for automated AD diagnosis considered in our literature review, followed by some recommendations to improve the accuracy and reliability of early AD diagnosis and facilitate monitoring of patients with AD. This information provides insight into important aspects of AI, including Trustworthy and Responsible AI. Developing Trustworthy and Responsible AI systems is fundamental to ensure that AI-based systems are reliable, safe, and ethical. The recommendations provide information about the possible solutions that can be used to fill the gaps in automated AD diagnosis. Considering these recommendations would contribute to the improved performance and reliability of automated AD detection systems, allowing medical professionals, patients, and other stakeholders to trust computer-aided diagnosis systems for more accurate and time-efficient disease diagnosis.

### 4.1. Challenges

Data scarcity – In spite of accurate and efficient performance of ML/DL models for CAD, fundamental challenges such as insufficient annotated data, that affects model generalization, limits their translation for clinical trials, due to patient privacy concerns, limited data availability, and labeling. To alleviate this problem, data augmentation and generation as well as transfer learning can be applied [ 37 ].

Biased data – During data generation, ethnicity or social background of engineers or medical professionals in charge can lead to biased dataset. Biases related to missing data information due to prevalence of a certain population is another type of bias in data. When the model is trained based on data from a specific geographical region, it might not perform well for people in other regions. Selection bias is another type of data bias, wherein, selected samples in research do not represent the entire population. Data heterogeneity is another type of bias. Indeed, various specific properties are considered during data generation, such as subject selection rules (ethnicity, gender, age, etc.), data acquisition protocols, and data annotation, which can lead to biased results.

Anonymization – Sharing information between institutions raises concerns about personal privacy. Removing sensitive and personal information of participants is of utmost importance. Another solution is FL, wherein data that is used for training remains in its original location.

Imbalanced data – Unequal distribution of classes in the training data can lead to bias and affect the performance of the model. For example, the Kaggle dataset suffers from this problem. Data augmentation, which is capable of increasing data in the minority class by a larger percentage than the majority class, can be applied to alleviate this problem [ 29 ].

Missing information – One of the main challenges when developing a CAD system is missing information in datasets, especially for sensor data and EMR. For example, in longitudinal studies, wherein records of each patient's baseline visit and multiple follow-up visits are available, not every test is performed at every visit. One solution in EMR data type is to consider each patient's data as a time series, providing a mechanism to fill in missing values in the records of patients [ 6 ].

Explainability – The black box nature of AI systems, especially DL models, has presented challenges with regards to model trustworthiness. Explainability refers to model transparency and the ability of a model to explain why and how a decision is reached. One solution to develop transparent DL models is to add explanation methods to the models, such as Saliency map and Class Activation Mapping (CAM) [ 2 , 17 , 20 , 23 , 24 , 30 ].

Lack of standardization – One of the main challenges when developing ML/DL models, especially for medical diagnosis, is lack of standardization. This includes variability in measurement techniques, evaluation methods, data collection and assessment, and data preprocessing techniques, among others. Applying different sources of data, such as neuroimaging data and non-imaging biomarkers, could result in higher performance of the model. However, variability in the nature of various data types provides challenges for fusing different sources of data [ 37 ]. For example, variability in neuroimaging data, that is 3D, and EMR, that is 1D, causes fusing of these two modalities challenging [ 35 ]. Further, unavailability of some modalities for some subjects due to incomplete data poses challenges [ 7 ]. The performance of CAD systems depends on the quality of data, which highlights the importance of data preprocessing. However, lack of standardized methods for image preprocessing across different datasets has presented challenges. For example, various preprocessing methods are applied to brain scans in different datasets, which makes the comparison between model performance of studies using different datasets unfair. In addition, in some studies, different datasets are used for development and model generalization. Differences in datasets, regarding data format, preprocessing stages, and image acquisition protocols, hinder accurate results. Even comparing studies that use the same dataset and the same number of subjects is not accurate because of differences in the fraction of data used for training and testing [ 7 ].

Feature Extraction – In medical imaging, capturing ROIs with the highest information for AD diagnosis needs domain-specific knowledge and it may neglect possible pathological locations and global structural information for accurate AD detection. On the other hand, global feature-based methods require high-dimensional data processing, especially for 3D images, which is time-consuming and computationally intensive and might neglect regional information that is important for AD diagnosis. In addition, due to the high dimensionality of features as compared with the small number of brain scans, chances are the model suffers from over-fitting. To overcome these challenges, some studies applied methods to extract and combine local, global, and spatial features from brain images for accurate AD diagnosis [ 2 ].

### 4.2. Future Perspectives and Recommendations

In the context of AD detection, diagnosing early MCI patients and predicting MCI to AD conversion are of more importance than other classification tasks [ 8 ]. Longitudinal studies, such as ADNI, provide more detailed information about signs of early MCI in the brain. To develop more efficient CAD systems for AD detection, data preprocessing, data augmentation, and fusion of multi-modal data should be considered [ 7 ]. In the medical imaging domain, patch-based and ROI-based methods are more efficient for AD classification because they are more sensitive to small abnormalities in the brain. Due to data scarcity, there is a need for developing data generation methods to generate missing data of a modality using another modality, for example generating PET scans from MRI [ 7 ].

Unintended biases or misalignment in datasets between training and deployment can adversely affect the performance of models. Currently, there is no standardized process for documenting ML datasets. Therefore, providing datasheets for datasets is of paramount importance to enhance transparency and mitigate biases in ML models. The key information to consider when documenting datasets are motivation, composition, collection, preprocessing/cleaning/labeling, applications and limitations, distribution, and maintenance. This information will allow dataset creators to carefully reflect on their dataset and consider the underlying assumptions, potential risks, and usage implications. For the dataset consumers, this will provide them with sufficient information to make informed decisions about utilizing a dataset [ 38 ].

The integration of EEG and other physiological sensor data with imaging data may offer a more dynamic and comprehensive understanding of the disease, aiding in the early detection and monitoring of AD progression. However, this multimodal approach is still in its infancy, with significant research required to standardize signal acquisition, processing protocols, and the fusion of disparate data types. Wearable technology, such as smartwatches and fitness trackers, can provide valuable data on daily activities, sleep patterns, and even physiological parameters like heart rate variability, which may correlate with disease progression or severity. However, research in this area is not as extensive as in medical imaging. There is a need for developing standardized protocols for data collection, preprocessing, and analysis specific to wearables. Research should be directed towards the development of ML models that can effectively process and analyze the high-dimensional time-series data generated by these devices. Wearable devices collect sensitive health data continuously, thus, addressing the challenges of data privacy and security is crucial. Exploring novel biomarkers that can be monitored through wearables, contributing to the early detection of AD or the transition from MCI to AD, is of utmost importance. Future research should also explore the integration of wearable sensor data with traditional imaging techniques to create a more holistic view of the disease. For example, changes in movement patterns detected by wearables could be paired with neuroimaging markers to refine the accuracy of early diagnosis. This multi-modal approach could improve progression monitoring and patient's response to treatment.

## 5. Conclusion

We performed analysis of recently published papers in AD detection based on various data modalities, such as signals and sensors, medical imaging, and EMR, to a total of 30 papers. Our comprehensive review was focused on the preprocessing stages (tailored to each data type), different learning methods, and evaluation metrics, along with a detailed explanation of the main publicly available datasets used for AD detection and their characteristics. We investigated potential research gaps, challenges, and opportunities in the context of automated AD detection.

Most automated AD detection studies are based on medical imaging and especially on MRI. This may be due to the availability of public imaging datasets for AD detection. In contrast, there exist few public datasets containing signals and sensors and EMR data types. Wearable sensors capture continuous, real-time data. Researchers can benefit from the integration of biomedical signals with imaging data, which will result in improved and early diagnosis as well as dynamic and comprehensive monitoring of patients with AD.

## Footnotes

1
https://adni.loni.usc.edu/

2
https://aibl.org.au/

3
https://www.kaggle.com/legendahmed/alzheimermridataset/metadata

4
https://sites.wustl.edu/oasisbrains

5
https://www.ucl.ac.uk/drc/research-clinical-trials/minimal-interval-resonance-imaging-alzheimers-disease-miriad

6
https://dementia.talkbank.org/bank.org/
